# Emerging Fungi and Diagnosis of Fungal Infections: Current Knowledge and New Developments

**DOI:** 10.3390/jof7040316

**Published:** 2021-04-19

**Authors:** Birgit Willinger

**Affiliations:** Division of Clinical Microbiology, Department of Laboratory Medicine, Medical University of Vienna, Waehringer Guertel 18-20, 1090 Vienna, Austria; birgit.willinger@meduniwien.ac.at

I would like to thank all the authors contributing to this Special Issue. I am extremely happy and thankful that 23 papers including reviews, communications and original articles of high quality were received for publication.

Diagnosing fungal infections, especially invasive fungal infections, is still challenging, as symptoms are not very specific and are often difficult to distinguish from bacterial or viral infections [1]. Additionally, emerging fungi which are difficult to detect or to identify hinder a timely and easy diagnosis [2]. However, early diagnosis is essential, as delaying treatment initiation is associated with an increased mortality. Conventional diagnostic methods for invasive fungal infections are each associated with shortcomings and therefore none of the currently available tests provide sufficient sensitivity and specificity alone; thus, the optimal approach relies on a combination of various testing strategies. This underscores the need for a constantly expanding knowledge of the variety of different pathogenic fungi, as well as the development of new techniques for the detection of fungal pathogens.

Articles in this Special Issue deal with different aspects of emerging fungi and their pathogenic role. Reviews dealing with the genus *Fusarium*, an emerging group of filamentous fungi, show different aspects of the medical relevance and emphasize its role concerning a One Health perspective [3,4]. *Scedosporium* and *Lomentospora* are also increasingly recognized pathogens. As they are resistant to many antifungal agents, it is important to know how to detect these fungi and enable a reliable and early diagnosis [5]. Mucorales and mucormycosis are emerging and may cause serious and life-threatening infections, requiring a rapid and reliable early diagnosis for a better outcome [6]. In addition, fungal infections in patients suffering from cystic fibrosis are expanding and remain challenging in diagnosis and treatment [7].

Not only invasive, but also local, deep or superficial infections such as vulvovaginal candidosis or dermatophytosis may be challenging, especially when caused by resistant fungi [8,9]. Terbinafin-resistant *Trichophyton mentagrophytes*—recently described in patients from India—seems to be on the rise and might be an important public health issue as transmission from the Indian subcontinent to Europe has already been described [10]. Facts and respective thoughts related to these topics can be read in this Special Issue’s excellent reviews. 

Improved standards of care depend on the development of new laboratory diagnostic and imaging procedures. The review discussing advances in in vivo molecular imaging shows new and promising approaches for the diagnosis of invasive pulmonary aspergillosis, and will direct future research [11]. 

The evolution of molecular tools for the detection of fungal pathogens has been slow in the last decades, but has significantly advanced in recent years. Several of the reviews and articles deal with the diagnostic repertoire of the mycology laboratory and show the value of new and well-established tests [12,13]. 

All articles in this Special Issue have added to the increasing knowledge of common and emerging fungi and their pathogenic role, as well as a better understanding of established or newly developed approaches for diagnosis. I wish to thank all authors and reviewers for their significant and highly appreciated contributions to this Special Issue, and for making it a valuable collection of studies which is very interesting to read and to learn from.

## References

[1] Schelenz S., Barnes R.A., Barton R.C., Cleverley J.R., Lucas S.B., Kibbler C.C., Denning D.W., British Society for Medical Mycology (2015). British Society for Medical Mycology best practice recommendations for the diagnosis of serious fungal diseases. Lancet. Infect. Dis..

[2] Lockhart S.R., Guarner J. (2019). Emerging and reemerging fungal infections. Semin. Diagn. Pathol..

[3] Hof H. (2020). The Medical Relevance of Fusariumspp. J. Fungi.

[4] Sáenz V., Alvarez-Moreno C., Pape P.L., Restrepo S., Guarro J., Ramírez A.M.C. (2020). A One Health Perspective to Recognize Fusarium as Important in Clinical Practice. J. Fungi.

[5] Chen S.C., Halliday C.L., Hoenigl M., Cornely O.A., Meyer W. (2021). Scedosporium and LomentosporaInfections: Contemporary Microbiological Tools for the Diagnosis of Invasive Disease. J. Fungi.

[6] Skiada A., Pavleas I., Drogari-Apiranthitou M. (2020). Epidemiology and Diagnosis of Mucormycosis: An Update. J. Fungi.

[7] Renner S., Nachbaur E., Jaksch P., Dehlink E. (2020). Update on Respiratory Fungal Infections in Cystic Fibrosis Lung Disease and after Lung Transplantation. J. Fungi.

[8] Sustr V., Foessleitner P., Kiss H., Farr A. (2020). Vulvovaginal Candidosis: Current Concepts, Challenges and Perspectives. J. Fungi.

[9] Berlin M., Kupsch C., Ritter L., Stoelcker B., Heusinger A., Gräser Y. (2020). German-Wide Analysis of the Prevalence and the Propagation Factors of the Zoonotic Dermatophyte Trichophyton benhamiae. J. Fungi.

[10] Gunzer M., Thornton C.R., Beziere N. (2020). Advances in the In Vivo Molecular Imaging of Invasive Aspergillosis. J. Fungi.

[11] White L.P., Price J.S. (2021). Recent Advances and Novel Approaches in Laboratory-Based Diagnostic Mycology. J. Fungi.

[12] Pasic L., Goterris L., Guerrero-Murillo M., Irinyi L., Kan A., Ponce C.A., Vargas S.L., Martin-Gomez M.T., Meyer W. (2020). Consensus Multilocus Sequence Typing Scheme for Pneumocystis jirovecii. J. Fungi.

[13] Camp I., Spettel K., Willinger B. (2020). Molecular Methods for the Diagnosis of Invasive Candidiasis. J. Fungi.

